# Multi-agent modeling of the South Korean avian influenza epidemic

**DOI:** 10.1186/1471-2334-10-236

**Published:** 2010-08-10

**Authors:** Taehyong Kim, Woochang Hwang, Aidong Zhang, Surajit Sen, Murali Ramanathan

**Affiliations:** 1 Departments of Computer Science and Engineering, State University of New York at Buffalo, 201 Bell Hall, Buffalo, NY 14260-1200, USA; 2 Departments of Physics, State University of New York at Buffalo, 239 Fronczak Hall, Buffalo, NY 14260-1500, USA; 3 Departments of Pharmaceutical Sciences, State University of New York at Buffalo, 427 Cooke Hall, Buffalo, NY 14260-1200, USA

## Abstract

**Background:**

Several highly pathogenic avian influenza (AI) outbreaks have been reported over the past decade. South Korea recently faced AI outbreaks whose economic impact was estimated to be 6.3 billion dollars, equivalent to nearly 50% of the profit generated by the poultry-related industries in 2008. In addition, AI is threatening to cause a human pandemic of potentially devastating proportions. Several studies show that a stochastic simulation model can be used to plan an efficient containment strategy on an emerging influenza. Efficient control of AI outbreaks based on such simulation studies could be an important strategy in minimizing its adverse economic and public health impacts.

**Methods:**

We constructed a spatio-temporal multi-agent model of chickens and ducks in poultry farms in South Korea. The spatial domain, comprised of 76 (37.5 km × 37.5 km) unit squares, approximated the size and scale of South Korea. In this spatial domain, we introduced 3,039 poultry flocks (corresponding to 2,231 flocks of chickens and 808 flocks of ducks) whose spatial distribution was proportional to the number of birds in each province. The model parameterizes the properties and dynamic behaviors of birds in poultry farms and quarantine plans and included infection probability, incubation period, interactions among birds, and quarantine region.

**Results:**

We conducted sensitivity analysis for the different parameters in the model. Our study shows that the quarantine plan with well-chosen values of parameters is critical for minimize loss of poultry flocks in an AI outbreak. Specifically, the aggressive culling plan of infected poultry farms over 18.75 km radius range is unlikely to be effective, resulting in higher fractions of unnecessarily culled poultry flocks and the weak culling plan is also unlikely to be effective, resulting in higher fractions of infected poultry flocks.

**Conclusions:**

Our results show that a prepared response with targeted quarantine protocols would have a high probability of containing the disease. The containment plan with an aggressive culling plan is not necessarily efficient, causing a higher fraction of unnecessarily culled poultry farms. Instead, it is necessary to balance culling with other important factors involved in AI spreading. Better estimations for the containment of AI spreading with this model offer the potential to reduce the loss of poultry and minimize economic impact on the poultry industry.

## Background

Several highly pathogenic avian influenza (AI) outbreaks have been reported over the past decade. According to a report of the World Health Organization (WHO) issued in December 2005, the threat of an influenza pandemic occurring in the near future has been exacerbated with the recent appearance and widespread distribution of the avian influenza virus H5N1 [1]. In July 2009, the Ministry of Health of Egypt reported three newly confirmed human cases of avian influenza A (H5N1) [2]. Of the 81 cases confirmed in Egypt to date, 27 have been fatal. The report warned that the next pandemic could result in the deaths of at least 2 to 7 million people, with tens of millions requiring medical attention, in the best-case scenario.

Avian influenza A (subtype H5N1) has already caused widespread outbreaks among poultry in Southeast Asia, with sporadic transmission from birds to humans (5) and limited probable human-to-human transmission [3-6]. South Korea recently faced H5N1 outbreaks whose economic impact was estimated to be 6.3 billion dollars, equivalent to nearly 50% profit generated by the poultry-related industries in 2008.

AI causes a contagious infection in domesticated birds including chickens, ducks, and turkeys. The infections take two main forms that are distinguished by low and high virulence. The "low pathogenic" form may go undetected because it causes mild symptoms, e.g., ruffled feathers and a drop in egg production. The highly pathogenic form spreads more rapidly with a disease course that affects multiple internal organs; its mortality rate can reach 90-100% within 48 hours [7-9]. The virus is excreted in the feces and secretions from the nose, mouth and eyes of infected birds. Contact with infected fecal material is the most common means of bird-to-bird transmission but AI can also spread by direct contact between healthy and infected birds and via airborne secretions.

Although the risk of AI of humans is low, confirmed cases of human infection from several subtypes of AI infection have been reported since 1997 in Asia and parts of Europe, the Near East and Africa [10,11]. The human cases have occurred predominantly in previously healthy children and young adults with direct or close contact with H5N1-infected poultry. Bird flu has killed more than 150 people worldwide since late 2003 and the human mortality has been approximately 50%.

There is little immune protection against AI in the human population because the virus generally does not infect humans. Furthermore, because influenza viruses have the ability to change, there is a looming possibility that AI could gain the capacity for more efficient human-to-human transmission [12-14]. A worldwide influenza pandemic in humans could occur under these circumstances. In June 2009, the World Health Organization declared a worldwide pandemic of influenza that was caused by a "swine flu" H1N1 virus, containing genetic elements from human, pig and bird influenza viruses [15].

Efficient control of potential influenza pandemics could be an important strategy in minimizing its adverse economic and public health impacts [16]. Mathematical models can be useful for identifying critical parameters for public health intervention strategies in pandemics and to elucidate the underlying complexity and nonlinear relationships [17-19]. Simulation studies have been used to predict temporal and spatial epidemic spreading patterns [14,20-26]. A contact pattern model of smallpox spread found that outbreaks could be contained by targeted vaccination combined with early detection without resorting to mass vaccination [3]. A stochastic epidemic model has been used to evaluate the effectiveness of targeted antiviral prophylaxis, quarantine, and pre-vaccination in containing an emerging influenza strain [4]. In addition to avian influenza (H5N1), influenza A (H1N1) virus has spread rapidly across the world [27,28]. Several reports have analyzed virus spreading patterns and effective vaccination strategies for maximizing the H1N1 containment [15,29].

Efficient control of AI outbreaks could be an important strategy in minimizing its adverse economic and public health impacts. This paper focuses on the results of numerical experiments with a stochastic multi-agent dynamics model with data obtained from an AI outbreak in South Korea in April 2008. This work is organized as follows. In the following model section, we describe the spatial distribution of chicken and duck flocks in poultry farms in South Korea and provide an overview of the trajectory of the 2008 South Korean AI outbreak. We also describe the stochastic dynamics model and discuss the methodology of our numerical experiments. We present the results of this study in five sub-sections that analyze the critical properties associated with AI outbreak spreading.

## Methods

### Distribution of Poultry and Poultry Farming in South Korea

We obtained relevant data sets by requesting information from several South Korean organizations including the Ministry for Food, Agriculture, Forestry and Fisheries, Korea Duck Association and the Korea Poultry Association [30].

Poultry farming is an important component of the agricultural economy of South Korea with an economic value estimated at nearly $600 million. More than 98 percent of chicken poultry farms and 93 percent of duck poultry farms are of small (< 2,000 chickens or < 500 ducks) or medium (2000-9999 chickens or 500-1,999 ducks) sized. However, the large commercial-scale facilities (≥ 10,000 chickens or ≥ 2000 ducks) account for more than 93 percent of the total number of chickens and more than 97 percent of the total number of ducks.

**Figure 1:**
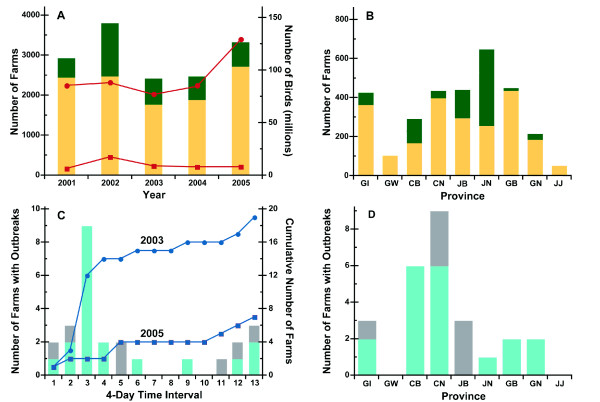
**A, Large size chicken (yellow bars) and duck (green bars) farms and their respective populations of chickens (red circles) and ducks (red square) in South Korea by year are shown**. B, Spatial distribution of chicken (yellow bars) and duck (green bars) farms in South Korea by province are shown. C, The number of AI outbreaks in the poultry farms in South Korea 2003 (blue bars) and 2005 (gray bars) are shown after the first outbreak. Bars represent the number of outbreaks during the specified period. The line represents the cumulative number of AI outbreaks in poultry farms in South Korea 2003 (blue circle) and 2005 (blue square). D, The number of outbreaks on poultry farms is shown by the provinces in South Korea 2003 (blue bars) and 2005 (gray bars). There is no AI outbreak in GW and JJ province due to geographical reasons. Specifically, GW province is the province farthest away from the first AI outbreak and JJ province is an island of South Korea, which can hardly or cannot be directly reached by truck transportation.

The statistics of chicken and duck poultry from large sized farms in South Korea between 2001 and 2005 are summarized in Figure 1A. During this period, there were an average of 2,231 and 808 large sized chicken and duck farms and the average number of chickens and ducks housed were 92,640,000 and 10,129,000, respectively. Figure 1B summarizes the spatial distribution of the farms by province.

There have been several AI epidemics in South Korea. The 30% decrease in the number of poultry farms in 2003 compared to 2002 (Figure 1A) is attributable to the AI outbreaks in 2003. The time course and the provinces affected by the 2003 and 2005 epidemics are summarized in Figure 1C and 1D.

### Spatiotemporal Dynamics of the 2008 South Korean AI Epidemic

**Figure 2:**
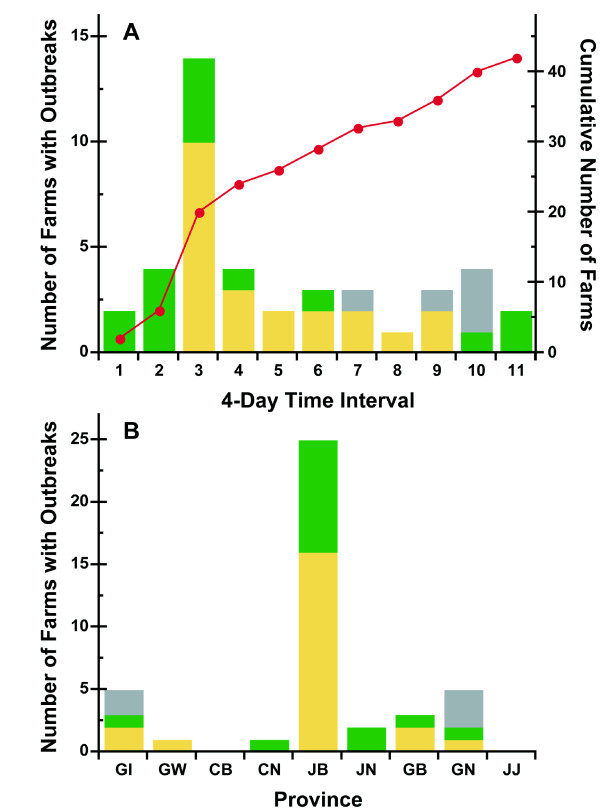
**A, The number of AI outbreaks in the poultry farms is shown after the first outbreak in South Korea 2008**. Yellow bars represent the number of outbreaks by chickens and Green bars represent the number of outbreaks by ducks. The line represents the cumulative number of AI outbreaks in poultry farms based on the outbreak reports by Ministry for Food, Agriculture, Forestry and Fisheries in South Korea 2008 in which the authorities instituted a quarantine in the area. B, The number of outbreaks on poultry farms is shown by the provinces in South Korea 2008. Green, yellow and gray bars represent the number of AI outbreaks of chicken, duck, and chicken-duck poultry farms.

The time course and spatial distribution of the 2008 outbreak are summarized in Figures 2A and 2B. Most of the AI outbreaks in 2008 occurred in Junbook province. The first AI outbreak occurred in Junbook province and it has most number of poultry and poultry farms. According to official reports, there were 43 AI outbreaks in 2008 following the initial outbreak, which was reported in Junbook province on April 1 2008. The second and third outbreaks occurred near the location of the first outbreak and were reported April 3 and 6, 2008, respectively. Four days after the first outbreak, Junbook province officials confirmed that the highly infectious H5N1 type of AI was responsible for the outbreak. There were 5 sequential outbreaks in Junbook province during the first week.

The problem became more critical on April 9, 2008 when an AI outbreak was reported in the Junnam province, and then in the Junbook province to its south. Subsequently, there were outbreaks in several other provinces including Chungnam, Gyungbuk and Gyungnam. AI spread to Gyunggi about a month after the first outbreak and eventually, by Day 44, most of the provinces in South Korea had reported outbreaks of the H5N1 infection.

South Korean authorities followed the AI containment recommendations of the World Health Organization (WHO) [11]. A special AI task force was convened. A containment area with a range of 3 km and a surveillance area with a range of 10 Km from the area of AI outbreaks were declared. All vehicles for transportation of chickens and ducks were sterilized. Sample chickens and ducks were obtained from poultry farms in the range of surveillance area and when AI infected samples as were found all chickens and ducks in those poultry farms were culled. All chickens and ducks in the range of surveillance area were restricted from being traded the public poultry markets.

### Modeling

We constructed a spatially explicit simulation of 103 million chickens and ducks in 3,039 poultry farms in South Korea. The model explicitly incorporates interactions among flocks of chicken and duck poultry, as these are known to be the primary contexts of influenza transmission and control measures can readily target locations such as farms and markets where large numbers of poultry are present. Random contacts among flocks of poultry birds from different farms are typically associated with day-to-day relocation by trading in public markets. The model does not consider the heterogeneity of poultry populations because poultry population data by poultry farm are lacking.

**Figure 3:**
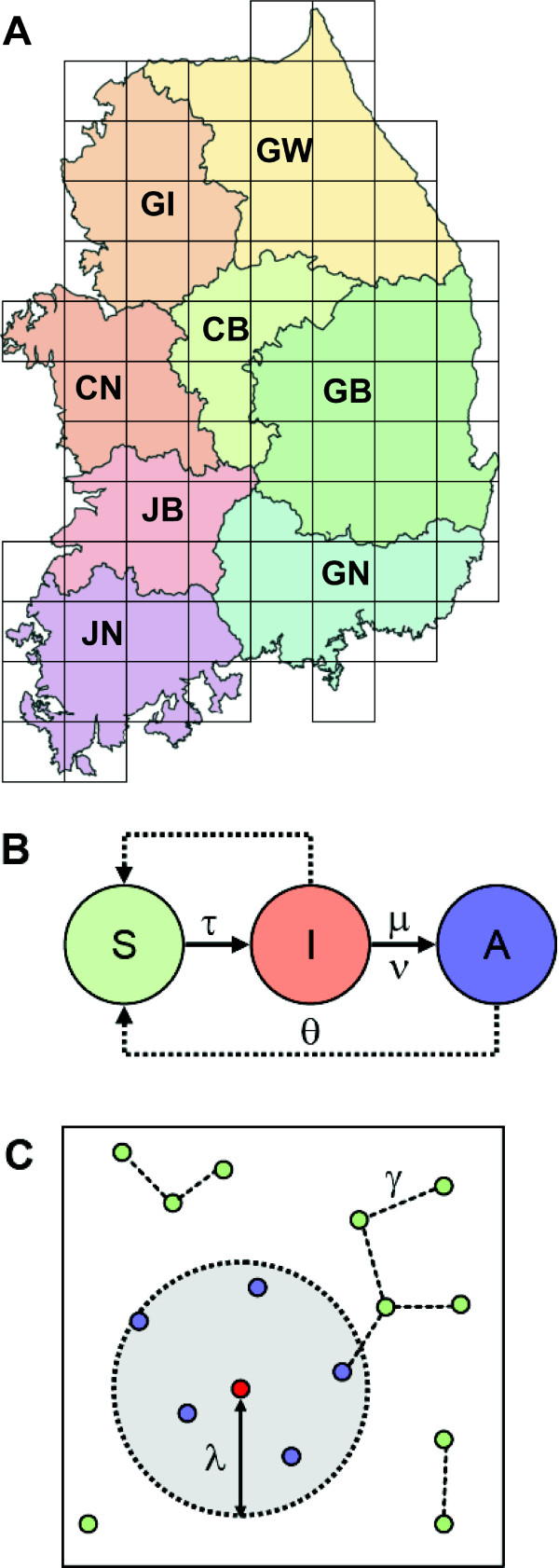
**A, The schematic representation of the model used in our simulations**. The map shows the land area of South Korea segmented into 76, 1 × 1 unit squares (1 unit *= *37.5 Km). The different color schemes represent the area of different provinces. B, Schematic representation of how an individual farm (a group of chickens or ducks) from the AI susceptible state (*S*) into incubation status (*I*) for the 2-17 day period (varies for chickens and ducks). In this status, a farm could transfer a virus without evidence of infection. After the period of incubation, it moves into activation status (A) where it could die by death probability of infected birds. C, γ is the infection spreading distance between poultry flocks and λ, is the radius of culling area from a center of infected poultry flocks.

Table 1 shows the abbreviations of each South Korean province and the number of square units occupied by each province. The number of chicken and duck poultry flocks in each province was based on the average the number of poultry and poultry farms reported in the census from 2001 through 2005 since data for 2006 and 2008 were not available to us. Data from eight provinces in South Korea were used in our simulations. We set up the population of poultry flocks in our model by region using 76 (37.5 km × 37.5 km) unit squares (Figure 3A) to form a spatial grid approximating the map of the South Korea. We introduced 3,039 poultry flocks representing 2,231 flocks of chickens and 808 flocks of ducks in this space. The groups of poultry flocks were randomly distributed within each province. We measured the transitions of the poultry population states by analyzing the interaction among poultry flocks for 50 time steps (1 time step *= *1 day).

**Table 1:** The number of blocks and the number of chicken and duck farms by province. The locations of the province are summarized in Figure 3A.

Province	Abbreviation on Map	Occupied blocks	Chicken farms	Duck farms
Gyunggi	GI	9	360	63
Gwangwon	GW	15	105	8
Chungbuk	CB	6	168	126
Chungnam	CN	7	420	35
Junbook	JB	6	294	144
Junnam	JN	10	260	390
Gyungbuk	GB	15	435	15
Gyungnam	GN	9	189	27
Jaeju (island)*	JJ	0	53	6

* The number of poultry farms is not included in the model since Jaeju is an island.

Figure 3B gives a schematic of the model used in our studies. The model parameters are succinctly summarized in Table 2. The parameters of our multi-agent model are relatively straightforward to interpret in the context of AI. The infection probability, τ, is a key factor that was used to model the spreading of the disease. A higher value of τ implies faster and easier transmission of AI among poultry. The value of τ was estimated based on the data available. Simulations were also conducted for four different infection rates: 0.1 (low), 0.3 (moderate), 0.6 (high) and 0.9 (extreme) for sensitivity analysis.

**Table 2:** Model parameters used for simulation.

Description	Parameter	Value
Size of unit square	**-**	37.5 km × 37.5 km
Infection probability	τ	1.0 (0.1, 0.3, 0,6, 0.9)
Chicken incubation period	μ	2.5 days (0-5 days)
Duck incubation period	ν	8.5 days (0-17 days)
Quarantine radius	λ	0.05 - 1.5 units
Long-range relocation probability	ω	0.0083 per day
Short-range relocation rate constant	*ψ*	162 units^-1^
Infection spreading distance	γ	0.15 unit
Death probability of infected birds	θ	0.5 per day
Delay in reporting outbreaks	ϕ	1 day (1, 2, 3) days

The parameters μ and ν are the mean incubation periods for chickens and ducks, respectively, and were obtained from values reported by both the World Health Organization and utilized by the South Korean Ministry of Food, Agriculture, Forestry and Fisheries during the AI outbreak [31]. The incubation period values in the simulation were assumed to be normally distributed random variates with mean μ and ν and with variance proportional to the square of the experimentally reported range of incubation period values. During the incubation period no control method could be applied to farms and the environment because the symptoms associated with the infection cannot be easily discerned. The lack of symptoms and/or human factors was assumed to cause a delay time of ϕ in reporting AI outbreaks. All parameters other than μ and ν were common to chicken and duck species in the model.

The infection spreading distance is represented by γ shown in Figure 3C. Farms within the infection spreading distance are risk of being infected by avian flu with an infection probability τ. The quarantine radius, λ, represents the range of the fixed radius culling area that is ordered for controlling spread of AI shown in Figure 3C.

Chickens and ducks are typically housed in poultry farms and there is not much spatial movement of the virus because of actual movement of the birds. However, chickens and ducks are moved in the course of trade. In the model, the movement of flocks of birds could be either local or long-range. We assumed that AI infected poultry flocks could not undergo movement. The probability of local movement was (1 - ω) and distance relocated was a random variate from an exponential distribution with rate constant ψ distance units^-1 ^(equivalently, the length scale is ψ^-1 ^distance units). The probability of long-range movement was ω and poultry flocks could be randomly relocated to any of the 76 unit squares that comprise South Korea in our system. Because we lacked information on the transportation routes for long-range movement and wild bird movement patterns in our model, we assumed that poultry flocks were relocated randomly.

The probability of an infected bird dying was represented by θ. We simulated the equivalent of 50 days of an AI outbreak. The average natural lifespan of chickens and ducks are 7-15 years and 10-20 years, respectively; however, the lifespan of in-house poultry can be as short as 45 days. In the model, we do not consider the natural death rate of the healthy poultry.

To obtain a parsimonious model of AI spreading, we let poultry flocks with an active AI infection in the functional radius λdie with probability τ. The spatial extent of AI spreading can then be written as:

$$\lambda_{t+\Delta t}=\lambda_{0}(1+f_{a}(t))\lambda_{t}\;\text{for}\;\text{time}\;t>0$$

where *f*_*a*_(*t*) is the ratio of the actual number of infected poultry flocks to the number of all poultry flocks in λ. In modeling AI spreading we set the infection probability, τ, of the infected poultry flocks within λ, which from a computing standpoint means that there is a stochastic process in *f*_*a *_as a function of *t *[32-34].

Although various other attributes and parameters could be applied to further enrich this model, the approach described provided a parsimonious yet effective model for describing the key spatiotemporal features of AI spread.

In each time step, the model entails three sequential processes, (i) onset of AI spreading (controlled by τ), (ii) warning messages to neighboring poultry farms and (iii) culling of poultry farms having high infection probability. We discuss (ii) and (iii) below.

When AI infection is detected, warning messages are distributed to all neighboring poultry flocks within a radius γ from the source. If the number of warning messages exceeds an actionable threshold, the culling process is activated; so that all birds in poultry farms in the range of λ are culled [35].

Let *q*_*i*_(γ) denote the number of poultry flocks *i *in the region of possible infection. We assign a weight 0 ≤ *w*_*k *_≤ 1 to characterize the state of fitness of a poultry flock, where 0 and 1 define death and best health, respectively. The infection level at any poultry flock *i *at the iteration step denoted via time *t*_*k *_can then be written as:

$$u_{i}(t_{k})=1-\sum_{k=1}^{q_{i}}w_{k}|q_{k}\geq 0$$

Observe that *u*_*i *_(0) = 0 (healthy) and a dead flock will have *u*_*i*_(*t *→ ∞) → 1.

It turns out that our model converges in time quickly to the final values of affected areas and hence the asymptotic behavior in *t *for our studies can be safely extracted. We can characterize the AI spreading via:

$$U(t)\equiv I(t)+C(t)\equiv \langle \frac{1}{2V_{0}}\sum_{i}^{}\sum_{t_{j}}^{t^{\prime }}u_{i}(t_{j})\rangle$$

where, *I*(*t*) represents the number of bird flocks affected by AI infection at time *t *and *C*(*t*) represents the culled bird flocks in farms at time *t*.

$$U(t\to \infty )\equiv \langle \frac{1}{2V_{0}}\sum_{i}^{}u_{i}(t\to \infty )\rangle$$

where, *V*_0 _is the total number of poultry flocks and the angular parenthesis denotes the average over 10^3 ^independent runs on the same random network.

#### Parameter Estimation

Parameter estimates were obtained by minimizing the squared difference between the observed data from the 2008 South Korean epidemic and the calculated results from our model. The model calculations were averaged over 100 realizations. The minimization objective function *F*(**P**) was:

$$F(P)\equiv \sum_{i}{\left[y(P,s_{i},z_{i},t_{i})-x(s_{i},z_{i},t_{i})\right]}^{2}$$

The **P **in the objective function represents the vector containing the model parameters that are to be estimated. The quantity *x*(*s*_*i*_, *z*_*i*_, *t*_*i*_) represents the data point corresponding to each observation *i *wherein *s*_*i *_is the species (either chicken or duck) corresponding to the data point, *z*_*i *_is the spatial region corresponding to the data point and *t*_*i *_is time over which the data point was accrued The *y*(**P**, *s*_*i*_, *z*_*i*_, *t*_*i*_) is the calculated value from the model that corresponds to *s*_*i*_, *z*_*i*_, *t*_*i*_. Simplex minimization with the Nelder-Mead algorithm was employed to determine the parameter estimates at the optimum [36]. Java code for the minimization algorithm was obtained from [37].

## Results

### Simulations Comparing AI Spread With and Without Containment Strategies

The outcomes from the multi-agent model were strongly dependent on the control strategies deployed and the spreading characteristics of the epidemic in the context of its environment. The model exhibited characteristics of spatially limited spread when effective containment strategies were deployed and widespread spatial dispersion followed by self-limited termination when the containment strategy proved ineffective. The parameters used for the model simulations are summarized in Table 2.

**Figure 4:**
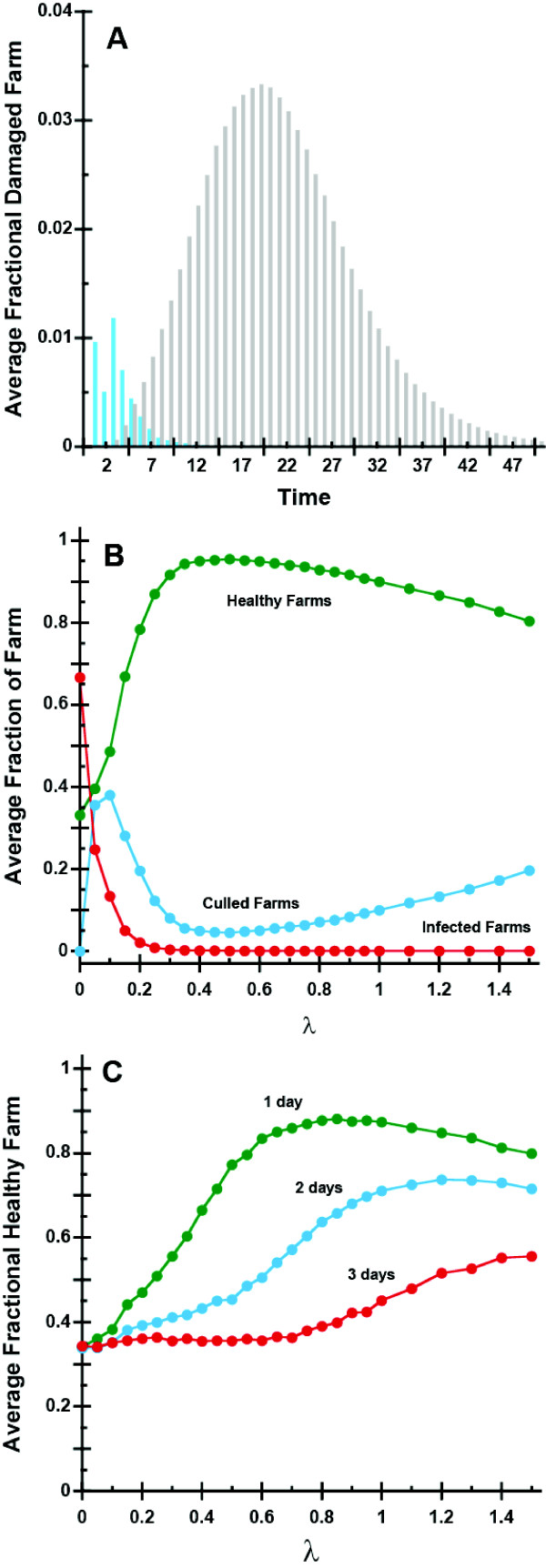
**A, The time course for the average fraction of culled and infected farms with (blue bars) and without the containment strategy (gray bars) are shown**. During the early stage of the AI infection, 0 to 10 days, there are a lot of culled birds in poultry farms in order to contain AI spreading. B, The average fraction of healthy (green circles), infected (red circles) and culled (blue circles) poultry farms at *t *= 50 days as a function of λ are shown. C, The adverse effects of reporting delays are shown. The average fraction of healthy farms at *t *= 50 days are plotted as function of λ. The curve with green circles, blue circles, and red circles represent ϕ = 1, ϕ = 2 and ϕ = 3.

Figure 4A shows the average time course from 10^3 ^AI outbreak simulation realizations of a large AI epidemic due to an initial outbreak at Junbook province in the absence and in the presence of the containment strategy. The extent of infection in the absence of a control strategy was significantly greater than that with a control strategy (Figure 4A). Two distinct processes cause loss of poultry in the model: death due to AI infection and death due to culling in the containment strategy. On average, when there is no containment strategy, the number of cases peaked 20 days after the initial outbreak. Upon occurrence of the first infection, the containment process initiated culling of about 1% of a flock of birds in poultry farms. The culling process prevents infection from spreading further to flocks of birds in neighboring poultry farms. The fraction of poultry flocks culled peaks on day 3; however, this culling reduces the loss of poultry at later times. Maximal damage occurred earlier in the presence of the control strategy primarily due to the culling process, whereas the damage in the uncontrolled epidemic scenario was entirely due to death by AI.

#### Efficient Containment Strategies

Figure 4B shows that there is an optimum value for the quarantine radius λ. Strategies involving a larger than optimum radius of culling or quarantine area cause unnecessarily culled flocks in poultry farms and strategies with a smaller than optimum radius of culling quarantine area cause loss of poultry due to dispersal of the AI infection.

Figures 4B shows the dependence of the average fractions of healthy poultry farms, poultry farms lost due to AI infection and culled farms as a function of λ. As λincreases, the fraction of healthy poultry farms increases until λ = 0.5. However, for λ > 0.5, the fraction of healthy farms decreases, which is caused by unnecessary culling. For λ < 0.1, the fraction of culled farms increases as λincreases. But for λ > 0.1, the fraction of culled farms decreases because culling prevents the infection from spreading to nearest available susceptible poultry. Therefore, the fraction of the healthy poultry farms keeps increasing until unnecessary culled farms are reported at the value of 0.5.

#### Effect of Reporting Delays

The extent of infection is strongly dependent on the reporting delays (Figure 4C). Rapid reporting and action is, as expected, a critical factor. Figure 4C shows the dependence of the average fraction of healthy poultry flocks on λ for reporting delay ϕ values of 1, 2, and 3 days. When outbreaks are reported within a day after AI symptoms appeared in the poultry flocks, the containment strategy works well (Figure 4C). However, a two-day delay in reporting the infection causes the containment strategy to be much less effective and three days of reporting delay substantially reduce the effectiveness of the containment strategy. These results show that early reporting dramatically affects the effectiveness of the containment strategy - early reporting is as important as deploying the optimal value of λ.

#### Effect of AI Incubation Period

**Figure 5:**
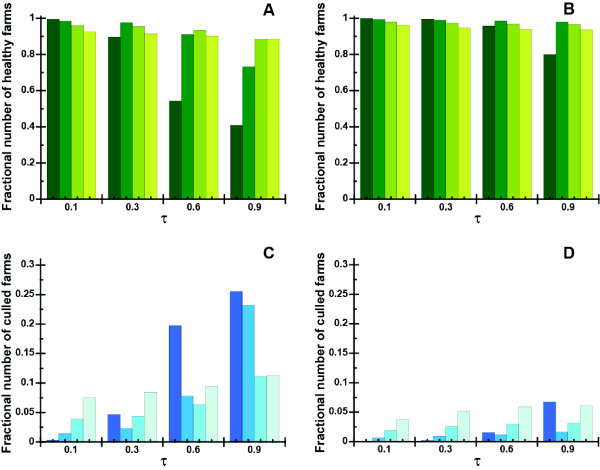
**The sensitivity of the different containment parameters are shown if containment efforts began 50 days after the first case**. Results of simulations are shown for different values of infection probabilities and incubation periods. Representative values of λ are 0.16 (dark green), 0.32 (green), 0.64 (light green) and 1.28 (yellow green) in Figures A and B, and 0.16 (dark blue), 0.32 (blue), 0.64 (light blue) and 1.28 (grayed blue) in Figures C and D. A, The fractional number of healthy birds in poultry farms as the function of τ is shown with normal chicken and duck incubation periods. B, The fractional number of healthy birds in poultry farms as the function of τ is shown without the incubation periods. C, The fractional number of culled birds in poultry farms as the function of τ is shown with normal chicken and duck incubation periods. D, The fractional number of culled birds in poultry farms as the function of τ is shown without the incubation periods.

Additionally, we conducted sensitivity analysis to examine the effect of AI incubation period because this parameter affects the ability to detect the presence of infected birds. Figures 5B and 5D were obtained with μ and ν = 0 whereas Figures 5A and C were obtained with the incubation periods of μ = 2.5 days for chickens and ν = 8.5 days for ducks as shown in the Table 2. A representative range of λ values and infection probabilities were examined. All the data were obtained at 50 days from the initial infection. The results demonstrate that containment is more effective in Figures 5B and D because the disease symptoms become manifest immediately upon infection and containment strategies can be implemented promptly.

The results in Figures 5A and 5C demonstrate that a nuanced approach to containment is necessary for AI that spreads with high infection probability (τ = 0.6 and τ = 0.9) and has a longer incubation period (μ = 2.5 days for chickens and ν = 8.5 days for ducks). Small λ values result in greater overall loss of poultry; in comparison, the results in absence of an AI incubation period are relatively insensitive to λ values over the entire range of infection probability values investigated.

### Modeling the Data from the 2008 South Korean Epidemic

We used least square regression to fit our model to the data and to estimate the model parameters corresponding to infection probability (τ), quarantine radius (λ), long-range relocation probability (ω), short-range relocation length scale (*ψ*), and incubation period for ducks (ν) for describing the data.

**Figure 6:**
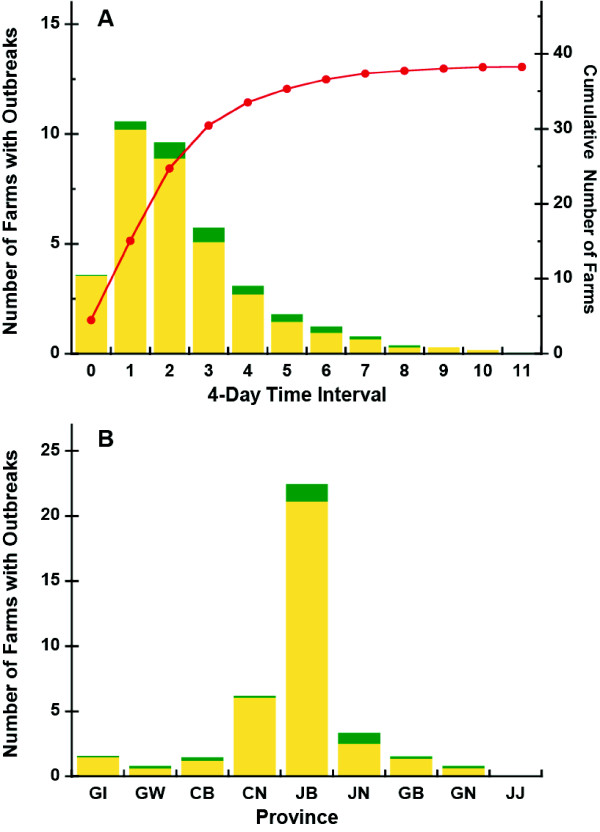
**Results from fitting our model to data based on the 2008 South Korea AI epidemic**. A, The time course of AI outbreaks in chickens (yellow bars) and ducks (green bars) after an initial outbreak in Junbook province are shown. The line represents the cumulative number of AI outbreaks in poultry. B, The spatial distribution by province of AI outbreaks in chickens (yellow bars) and ducks (green bars) predicted by our model are shown. Green and yellow bars represent the averaged number of AI outbreaks of a flock of chickens and ducks in poultry farms. The data in both Figures are averaged over 100 simulations of the model.

The parameters estimated from the regression analyses are summarized in Table 3. The time course and spatial distribution predicted by the model are shown in Figure 6. Comparing to Figure 2, the model predicted an average of 39 outbreaks, which compares favorably with the 42 outbreaks that were reported by the South Korean authorities. The predicted time course had a maximum value at the first and second 4-day intervals. The model also predicted an increased number of outbreaks in Junbook compared to other provinces as observed in the 2008 South Korean AI epidemic. Although the simulated results from the modeling exhibited quantitative features similar to the data from the 2008 South Korean AI epidemic, the simulations tended to underestimate the occurrence of outbreaks in ducks and the simulated time course had less abrupt variations than those observed.

**Table 3:** Model parameters estimated from the 2008 South Korean epidemic.

Description	Parameter	Fixed or Estimated	Value
Size of unit square	**-**	Fixed	37.5 km × 37.5 km
Infection probability	τ	Estimated	0.429 day^-1^
Chicken incubation period	μ	Fixed	2.5 days
Duck incubation period	ν	Estimated	7.2 days
Quarantine radius	λ	Estimated	0.293 units
Long range relocation probability	ω	Estimated	0.0083 day^-1^
Short-range relocation rate constant	*ψ*	Estimated	162 units^-1^
Infection spreading distance	γ	Fixed	0.15 unit
Death probability of infected birds	θ	Fixed	0.5 day^-1^
Delay in reporting outbreaks	ϕ	Fixed	1 days

Based on the modeling, we estimated the value of λ for the 2008 South Korean AI epidemic would be λ = 0.293 which is equivalent to an 11 Km of quarantine radius. The λ value from our modeling corresponding to estimated parameters from the 2008 South Korean AI epidemic was λ = 0.465. From the modeling, we could analyze the efficacy of the South Korean authorities' strategies on AI outbreaks in 2008. We calculated the protection rate of poultry farms at time *t*, *R(t)*, on AI outbreak via:

$$R(t)=100-\left(\frac{I(t)+C(t)}{V_{0}}\times 100\right)$$

where, *I*(*t*) represents the number of poultry flocks affected by AI infection at time *t*, *C*(*t*) represents the culled poultry flocks at time *t *and *V*_0 _is the total number of poultry flocks at time 0. Our model estimated parameter values predict the protection of 96.5% of poultry at time *t *= 50 days. The South Korean authorities reported the protection of 94.3% of poultry. This result suggests the South Korean containment strategy was very effective given the epidemic conditions at controlling the 2008 AI outbreak.

**Figure 7:**
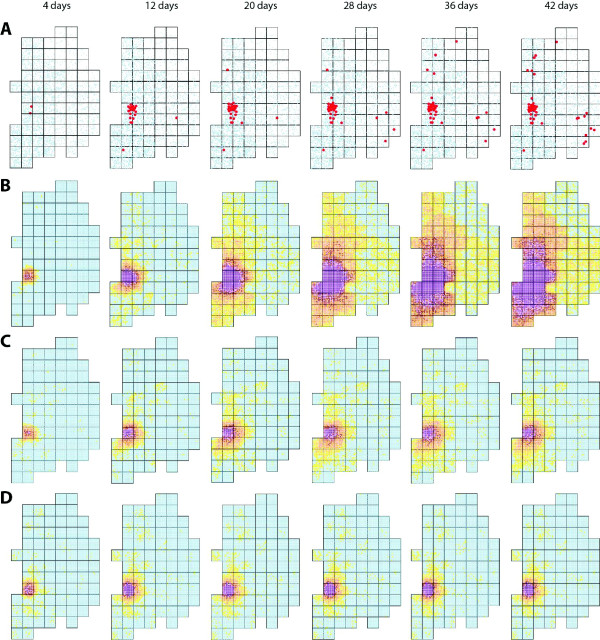
**Spatiotemporal dynamics of the AI outbreaks in South Korea in 2008 are shown at 4, 12, 20, 28, 36 and 42 days after the first outbreak**. Colors represents the infection intensity from low (yellow) to high (purple) for AI spreading including culling and infected poultry farms. A, Actual data for the 2008 AI epidemic from the Ministry of Food, Agriculture, Forestry and Fisheries, South Korea are shown. B, Simulation in the absence of a containment strategy is shown. C, Simulation from our model for parameter values of λ = 0.293 and τ = 0.429, which were selected to mimic the South Korea containment strategy is shown. D, Simulation with an estimated λ value of λ = 0.465 and τ = 0.429 is shown. The columns correspond to the times indicated. All simulation results are averaged over 100 simulations of the model.

The heat maps in Figure 7 compare the observed patterns of outbreaks during the 2008 South Korean AI epidemic to simulation results for the spatiotemporal dispersal of the AI epidemic for different containment scenarios. In the absence of containment, the model predicts that AI would spread to almost the entire country. Because of the initial outbreak occurred in Junbook (JB) province, the southwestern part would be most strongly affected. The northeastern part is less affected because of the lower density of poultry farms. The heat maps also highlight the potential improvements possible with the implementation of a containment strategy with an estimated value of λ = 0.465.

## Conclusions

We modeled a spatially explicit simulation of how AI spread in South Korea in 2008. The model explicitly incorporates interactions among flocks of chicken and duck poultry, as these are known to be the primary contexts of influenza transmission. Our study confirms that the analysis of spatiotemporal epidemic patterns has considerable power to help illuminate epidemic dynamics [20].

Our studies suggest that the containment plans with well chosen parameters are critical for minimizing loss of poultry flocks. The containment plans with aggressive quarantine areas λ > 0.5, are unlikely to be effective because they can result in a higher fraction of unnecessarily culled poultry flocks. The containment plans with smaller quarantine areas, λ < 0.5, are also unlikely to be effective, because they can result in a higher fractions of infected poultry flocks. In addition, reliable information about the important factors identified here along with an efficient and effective quarantine plan is needed to achieve high levels of containment. Although the effectiveness of most containment strategies can be good, any delays with regard to understanding the local-environment specific data could allow the pandemic to spread [3].

We also estimated parameters obtained by minimizing the squared difference between the observed data from the 2008 South Korean epidemic and the calculated results from our model. Data availability led us to model South Korea rather than any perceived greater risk of emergence compared to other countries in the region; however, we believe our conclusions may be potentially relevant to controlling the spread of AI in other parts in the world. If a newly emergent influenza strain appears, the model could be calibrated to the available data and used to determine reasonable intervention options at the epidemic source.

Currently the World Health Organization (WHO) tracks the number of avian-to-human and possible human-to-human transmission events reported [38]. The widespread distribution of the avian influenza virus H5N1 has increased the risk for the occurrence of an influenza pandemic in the near future [39]. The detection of even a single case of human-to-human transmission of the avian influenza virus could necessitate heightened pandemic alerts that have high economic cost to countries that are affected [40]. The overarching fear in public health circles is the possible occurrence of a flu strain with virulence comparable to the H1N1 virus of 1918, which killed nearly 40 million people [41]. Avian flu viruses are now common in domesticated poultry but despite the industrial scale of modern poultry farming, they have not gained traction in the human population [42,43]. Flu viruses however can undergo reassortment - indeed the ongoing 2009 H1N1 "swine flu" epidemic in the United States is a virus strain that has undergone reassortment. The possibility that a deadly form the H5N1 avian flu could mix with the highly transmissible H1N1 virus to make a virus with the worst properties of both cannot be precluded [44]. Our modeling approach provides a basis for using information from prior epidemics to develop containment strategies and evaluate what-if scenarios for evaluating their potential efficacy at different conditions.

To apply this model in other countries or different subtypes of a strain, the parameters we provided can be modified to meet different conditions and environments. However, this model did not sufficiently incorporate complicated environmental factors, such as effects of transportation routes, wild bird movement pathway, local weather and geographical characteristics. In addition, the stagnant population of poultry and the lack of attributes for different types of poultry farms could make the model limited when used for generalized AI spread simulation.

A few of the model parameters we have incorporated, such as infection probability, incubation periods, relocation probability and relocation ranges, could not be reliably ascertained from the fitting. For example, the range of latency period we have used, μ = 2.5 days and ν = 8.5 days, would not be long enough to simulate for low pathogenic AI (LPAI) since the latent period of LPAI could reach over 2 weeks. To reduce these uncertainties, prompt and accurate information obtained from high-quality epidemiological data by public health agencies around the world is critical to estimate the infection from virus spreads. Such improvement in our understanding of how a virus behaves will extend our options both to control avian influenza and our ability to predict virus spread by epidemics. Regardless of uncertainties, preparedness with this type of simulation would be extremely valuable in effective planning and modeling of oncoming disease outbreaks. A feasible strategy for containment of the spreading of AI offers the potential to reduce loss of poultry, minimize economic impact on the poultry industry, and prevent possible threats on the human population.

## Competing interests

The authors declare that they have no competing interests.

## Authors' contributions

The work presented in the article was carried out in collaboration between all authors. Specifically, TK and WH designed algorithms and implemented experimentation environments. MR supervised the research, designed key modeling strategies and worked on evaluation of results and revision of the manuscript. SS and AZ have supervised this research project. All authors read and approved the final manuscript.

## Pre-publication history

The pre-publication history for this paper can be accessed here:

http://www.biomedcentral.com/1471-2334/10/236/prepub
